# Spontaneous All-Sequence Baroreflex Sensitivity Across Predialysis Chronic Kidney Disease Categories: A Cross-Sectional Study

**DOI:** 10.7759/cureus.114284

**Published:** 2026-08-10

**Authors:** Abha Prakash, Amritesh Kumar, Kumar Abhishek, Sunita, Prit P Singh, Manish Kumar, Chandan Kumar, Tarun Kumar

**Affiliations:** 1 Physiology, Indira Gandhi Institute of Medical Sciences, Patna, IND; 2 Nephrology, Indira Gandhi Institute of Medical Sciences, Patna, IND

**Keywords:** autonomic dysfunction, baroreflex sensitivity, cardiovascular risk, chronic kidney disease, predialysis, sequence method

## Abstract

Introduction

Cardiovascular autonomic dysfunction contributes to the excess cardiovascular burden of chronic kidney disease (CKD). Spontaneous baroreflex sensitivity (BRS) quantifies the cardiac interval response to short-term changes in systolic blood pressure (SBP); however, balanced comparisons across consecutive predialysis CKD categories remain limited.

Materials and methods

This cross-sectional observational study was conducted from May 2024 to October 2025 in the Autonomic and Vascular Function Testing Laboratory, Department of Physiology, Indira Gandhi Institute of Medical Sciences (IGIMS), Patna, in collaboration with the Department of Nephrology. It used stage-stratified consecutive sampling to recruit 162 adults aged 18-65 years with predialysis CKD, with 54 participants in each of the G3, G4, and G5 categories. After 10 minutes of supine rest, a continuous five-minute stationary segment of simultaneous electrocardiographic and beat-to-beat arterial pressure signals was analyzed. Spontaneous all-sequence BRS was calculated using Nevrokard BRS version 6.3.0 (Nevrokard Kiauta, Izola, Slovenia). Category differences were assessed using Welch analysis of variance with Games-Howell pairwise comparisons. A post hoc multivariable linear regression sensitivity analysis using HC3 robust standard errors was performed to adjust for age, sex, body mass index (BMI), and resting SBP.

Results

The mean BRS was 10.87 ± 5.02 ms/mmHg in G3, 7.05 ± 3.39 ms/mmHg in G4, and 3.44 ± 1.86 ms/mmHg in G5. The overall difference was significant (p < 0.001), and all pairwise comparisons remained significant after adjustment for multiple comparisons. In an exploratory multivariable sensitivity analysis, BRS remained significantly lower in G4 and G5 compared with G3 after adjustment for age, sex, BMI, and resting SBP.

Conclusions

Spontaneous all-sequence BRS was substantially lower across successively higher predialysis CKD categories, and the association persisted after adjustment for age, sex, BMI, and resting SBP. However, the cross-sectional design does not establish temporal progression, causality, or prognostic utility.

## Introduction

Chronic kidney disease (CKD) is independently associated with substantial cardiovascular morbidity and mortality, and this excess risk becomes evident well before the initiation of dialysis. Although traditional cardiovascular risk factors contribute to this burden, they do not fully explain the disproportionately high incidence of arrhythmia, sudden cardiac death, heart failure, and vascular events observed in patients with impaired kidney function. CKD-specific disturbances, including retention of uremic toxins, oxidative stress, chronic inflammation, endothelial dysfunction, disordered mineral metabolism, vascular calcification, volume-related hemodynamic stress, sympathetic overactivity, and parasympathetic withdrawal, collectively create a high-risk cardiovascular and autonomic phenotype [1-4].

The arterial baroreflex is a rapid negative-feedback mechanism that stabilizes arterial pressure by continuously modifying cardiac and vascular autonomic outflow. Stretch-sensitive mechanoreceptors located predominantly in the carotid sinus and aortic arch detect short-term changes in arterial wall distension and transmit afferent signals to cardiovascular regulatory centers in the medulla. These central pathways subsequently adjust parasympathetic and sympathetic activity to the heart and peripheral vasculature. Cardiac baroreflex sensitivity (BRS) quantifies the change in cardiac interval produced by a unit change in systolic blood pressure (SBP) and is conventionally expressed in milliseconds per millimeter of mercury. A lower BRS indicates reduced capacity of the cardiovascular autonomic system to buffer acute blood-pressure fluctuations [4,5].

Impairment of cardiac BRS may be particularly relevant in CKD because several consequences of kidney dysfunction can affect both the neural and mechanical components of the baroreflex. Sympathetic overactivation, parasympathetic withdrawal, autonomic neuropathy, arterial stiffening, vascular calcification, endothelial dysfunction, and altered volume regulation may reduce the responsiveness of the reflex arc. Previous studies in predialysis CKD have demonstrated reduced cardiac BRS and associations with lower glomerular filtration rate, increased arterial stiffness, impaired vascular compliance, and adverse cardiovascular profiles [6-10]. These observations suggest that baroreflex dysfunction may represent an integrated physiological marker of autonomic and vascular impairment in CKD.

More recent investigations have extended this evidence across different kidney-disease populations and analytical approaches. Martinez-Alanis et al. demonstrated lower sequence-derived BRS in patients with end-stage renal disease than in healthy individuals and highlighted the influence of signal delay and sequence-selection methodology on BRS estimation [11]. Sabino-Carvalho et al. reported impaired sympathetic baroreflex control in patients with nondialysis CKD, providing complementary evidence that kidney dysfunction affects both cardiac and sympathetic components of the arterial baroreflex [12]. Theodorakopoulou et al. observed substantial impairment of BRS in both hemodialysis and peritoneal dialysis populations, although BRS did not differ significantly according to dialysis modality [13]. Improvement in BRS following renal transplantation suggests that at least part of the autonomic abnormality may be reversible, whereas uremic autonomic neuropathy provides an additional biological mechanism for persistent dysfunction [14,15].

Despite this accumulating evidence, balanced comparisons of spontaneous all-sequence cardiac BRS across consecutive predialysis CKD categories remain limited. Most previous studies have compared CKD with healthy controls, evaluated mixed CKD populations, focused on patients receiving dialysis, or used different BRS estimation methods. A category-wise analysis confined to predialysis CKD may therefore help determine whether cardiac baroreflex impairment is already distinguishable across progressively lower levels of kidney function before dialysis begins.

The objective of the present study was to compare spontaneous all-sequence cardiac BRS across CKD categories G3, G4, and G5 using a uniform physiological recording and analytical protocol.

## Materials and methods

Study design and setting

This cross-sectional observational study was conducted from May 2024 to October 2025 in the Autonomic and Vascular Function Testing Laboratory, Department of Physiology, Indira Gandhi Institute of Medical Sciences (IGIMS), Patna, in collaboration with the Department of Nephrology. Participants were identified and screened in the Nephrology Outpatient Department (OPD) of IGIMS, Patna, before initiation of maintenance dialysis. Eligible participants were subsequently referred to the Autonomic and Vascular Function Testing Laboratory, Department of Physiology, where the standardized physiological assessment and BRS recording were performed.

The Institutional Ethics Committee of IGIMS, Patna, approved the study (approval number 1538/IEC/IGIMS/2024). Written informed consent was obtained from every participant after the participant information sheet had been explained in a language the participant understood. The study was conducted in accordance with the Declaration of Helsinki. The study was not prospectively registered with the Clinical Trials Registry-India (CTRI), as it was conducted as a non-interventional cross-sectional observational study without allocation of any therapeutic intervention.

Participants, sampling, and CKD classification

Adults aged 18-65 years with a nephrologist-documented diagnosis of predialysis CKD G3, G4, or G5 were assessed for eligibility. The inclusion and exclusion criteria are summarized in Table 1. Stage-stratified consecutive sampling was used. Within each CKD category, eligible patients presenting consecutively to the Nephrology outpatient service were approached for participation until the prespecified target of 54 analyzable participants in that category was reached. No random sampling or random allocation was performed. Recruitment for a category stopped once its prespecified target was achieved.

**Table 1:** Inclusion and exclusion criteria BRS: baroreflex sensitivity; CKD: chronic kidney disease; KDIGO: Kidney Disease: Improving Global Outcomes

Criteria	Details
Inclusion criteria	Adults aged 18–65 years
	Nephrologist-documented diagnosis of predialysis CKD G3, G4, or G5
	CKD chronicity confirmed from nephrology records for at least three months according to KDIGO criteria
	Predialysis status before initiation of maintenance dialysis
	Written informed consent provided
Exclusion criteria	Maintenance hemodialysis
	Previous renal transplantation
	Pregnancy
	Refusal to consent
	Diabetes mellitus
	Established cardiovascular or cerebrovascular disease
	Current smoking
	Use of medications known to materially influence cardiac autonomic measurements
	Documented pre-existing diagnosis of hypertension or current antihypertensive treatment
	Persistent artifact, ectopy, or signal interference preventing reliable BRS analysis

An elevated blood pressure value recorded during the standardized study visit, in the absence of a prior clinical diagnosis or treatment history, was not independently used to exclude a participant.

CKD chronicity was confirmed from nephrology records documenting abnormalities of kidney structure or function for at least three months, in accordance with Kidney Disease: Improving Global Outcomes (KDIGO) criteria [16]. G3a (45-59 mL/min/1.73 m²) and G3b (30-44 mL/min/1.73 m²) were combined as G3; G4 was defined as 15-29 mL/min/1.73 m² and G5 as <15 mL/min/1.73 m² in participants not receiving dialysis. CKD categories G3, G4, and G5 were defined according to the estimated glomerular filtration rate (eGFR) documented in the clinical records.

The sample size was determined during study planning using G*Power software (Universitat Kiel, Germany) for a one-way ANOVA comparing three groups. Because no closely comparable category-wise study was available for deriving an anticipated effect size, a large standardized effect size (Cohen’s f = 0.50) was assumed, with a two-sided alpha level of 0.05 and 90% statistical power. This calculation yielded a minimum total sample of approximately 54 participants. To ensure adequate and equal representation of each CKD category and improve the precision of category-specific comparisons, the approved study protocol recruited 54 participants in each of the G3, G4, and G5 groups, resulting in a final planned sample of 162 participants.

Operational definitions and abbreviations

CKD categories were defined according to the eGFR: G3, 30-59 mL/min/1.73 m²; G4, 15-29 mL/min/1.73 m²; and G5, <15 mL/min/1.73 m² in participants not receiving maintenance dialysis. BRS was defined as the change in R-R interval associated with a unit change in SBP and was expressed in ms/mmHg. An “up sequence” represented concordant increases in SBP and R-R interval, whereas a “down sequence” represented concordant decreases in SBP and R-R interval. All-sequence BRS referred to the mean slope derived after combining all accepted up and down sequences. Diastolic blood pressure is abbreviated as DBP, body mass index as BMI, and confidence interval as CI. For eligibility, hypertension referred to a documented pre-existing clinical diagnosis or current antihypertensive treatment; an isolated elevated blood pressure recorded during the standardized study visit was not considered independently exclusionary.

Pre-test standardization and signal acquisition

Testing was performed in a quiet, temperature-controlled laboratory maintained at 22-25°C. Participants were instructed to avoid tea, coffee, tobacco or nicotine, and alcohol for at least 24 hours before testing, and recordings were obtained at least two to three hours after food intake. After a minimum of 10 minutes of supine rest, participants remained relaxed, silent, and motionless while breathing spontaneously. Respiratory frequency was not paced, and a dedicated respiratory channel was not recorded during the BRS acquisition. Continuous noninvasive beat-to-beat arterial pressure was recorded using the Human NIBP Nano system with wrist and height-correction units (Finapres Medical Systems, Amsterdam, the Netherlands). Electrocardiographic and arterial pressure signals were acquired simultaneously using a PowerLab 15T data-acquisition unit, model ML818, and LabChart Pro software (ADInstruments, Sydney, Australia). A continuous five-minute stationary, artifact-free segment with technically acceptable waveforms and without persistent ectopic interference was selected for BRS analysis. The investigator responsible for selecting the analysis segment was blinded to the participant’s CKD category at the time of segment selection. Spontaneous BRS was calculated using the sequence method in Nevrokard software with a minimum sequence length of three consecutive beats, a minimum SBP change of 0.5 mmHg, a minimum R-R interval change of 5 ms, a sequence correlation threshold of 0.85, and a one-beat delay between SBP and the corresponding R-R interval.

Spontaneous baroreflex sensitivity

**Figure 1:**
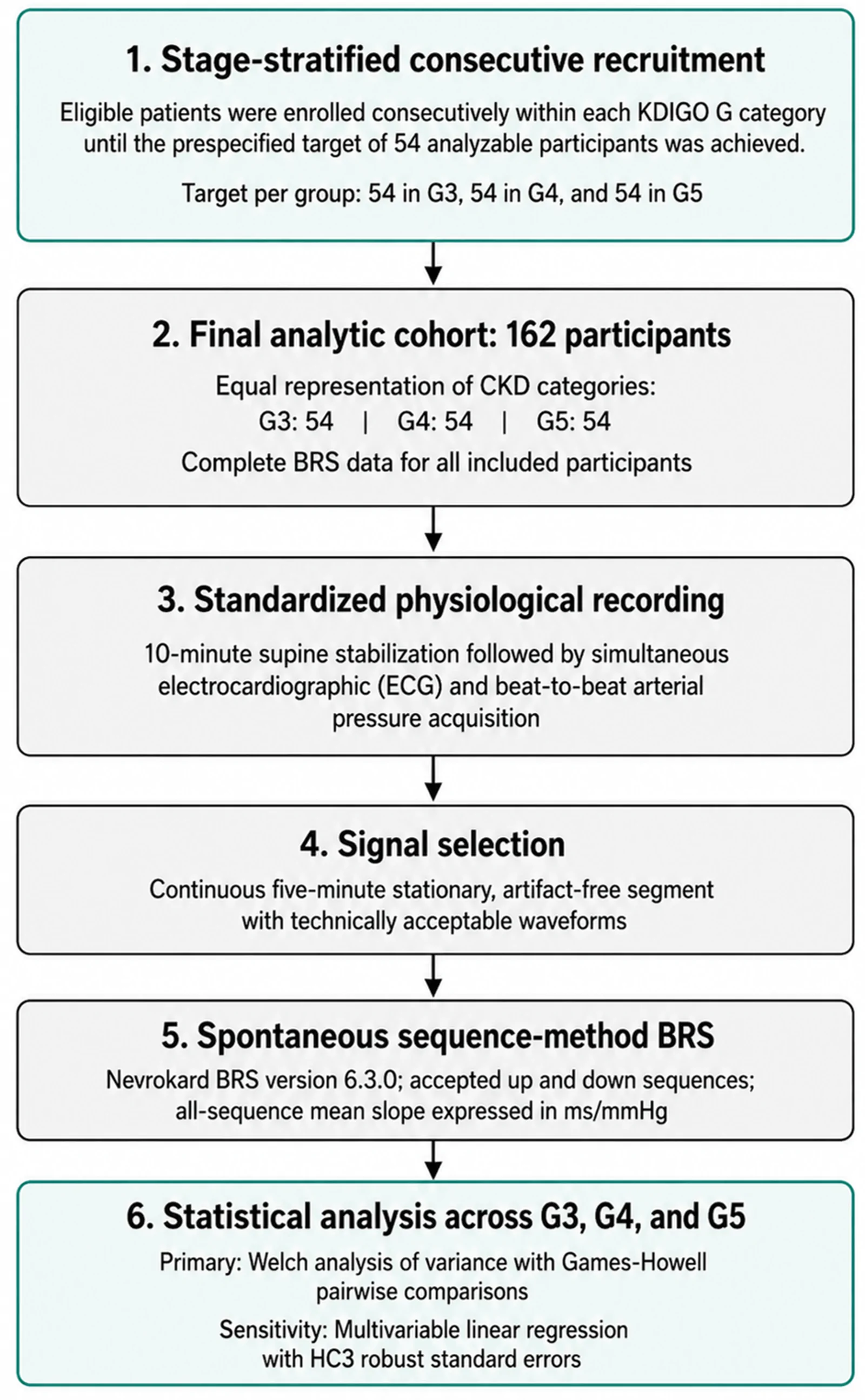
Participant selection and spontaneous baroreflex sensitivity analysis workflow Stage-stratified consecutive sampling was continued until 54 analyzable participants were enrolled in each CKD category, yielding a final cohort of 162 participants. Following standardized supine rest, a continuous five-minute artifact-free segment of simultaneously recorded electrocardiographic and beat-to-beat arterial pressure signals was analyzed using Nevrokard BRS version 6.3.0. The primary group comparison used Welch analysis of variance with Games-Howell pairwise comparisons, and the post-hoc sensitivity analysis used multivariable linear regression with HC3 robust standard errors. BRS: baroreflex sensitivity; CKD: chronic kidney disease; ECG: electrocardiography; HC3: heteroscedasticity-consistent standard error type 3

Spontaneous BRS was derived offline using Nevrokard BRS version 6.3.0 (Nevrokard Kiauta, Izola, Slovenia). The sequence method identified runs of at least three consecutive beats in which SBP and the corresponding RR interval changed concordantly. Increasing systolic pressure accompanied by RR-interval prolongation was classified as an up sequence, and decreasing systolic pressure accompanied by RR-interval shortening was classified as a down sequence. Linear regression was applied within each accepted sequence, and the slope was expressed in ms/mmHg. All-sequence BRS, the prespecified primary outcome, was the software-derived mean slope obtained after combining all accepted up and down sequences. Nevrokard BRS version 6.3.0 was used with standard/default sequence-method settings, including the documented minimum sequence length, systolic-pressure and RR-interval thresholds, correlation criterion, and lag structure. No user-defined modifications were made. The participant-selection and BRS-analysis workflow is summarized in Figure 1.

Statistical analysis

All statistical analyses were performed using IBM SPSS Statistics for Windows, version 26.0 (IBM Corp., Armonk, New York, United States). The final analysis included the prespecified balanced cohort, comprising 54 participants in each CKD category. Continuous variables are presented as mean ± standard deviation (SD), with 95% CIs where appropriate, and categorical variables as n (%). Distributional form was assessed using the Shapiro-Wilk test and variance homogeneity using Levene's test. Baseline continuous variables were compared using Welch analysis of variance, and sex distribution was compared using the chi-square test. BRS was compared using Welch analysis of variance because variance homogeneity was not satisfied; Games-Howell tests were used for multiplicity-adjusted pairwise comparisons. A conventional eta-squared effect size (η² = SSbetween/SStotal) was calculated descriptively from the between-group and total sums of squares. Because the primary inferential comparison used Welch ANOVA owing to heteroscedasticity, η² was not derived from the Welch F statistic and was interpreted as a descriptive estimate of the magnitude of the unadjusted category effect. An exploratory post hoc multivariable linear regression sensitivity analysis was subsequently performed with all-sequence BRS as the dependent variable, and CKD category entered as indicator variables, with G3 as the reference category. Age, sex, and BMI were included because each may influence cardiovascular autonomic function and BRS. Resting SBP was added in a second model because it differed significantly across CKD categories and may influence spontaneous BRS. eGFR was not entered because it defines CKD category and would therefore be collinear with the exposure classification. The regression analysis was intended as an exploratory sensitivity analysis rather than a prespecified confirmatory model. To account for heteroscedasticity, HC3 heteroscedasticity-consistent standard errors were used. Results are reported as unstandardized coefficients (B), robust standard errors, 95% CIs, and p-values. Multicollinearity was assessed using variance inflation factors. All tests were two-sided, and p < 0.05 was considered statistically significant. Reporting followed the Strengthening the Reporting of Observational Studies in Epidemiology (STROBE) statement [17].

## Results

Participant flow and baseline characteristics

Stage-stratified consecutive recruitment continued until the prespecified target of 54 analyzable participants was achieved in each CKD category. The final analytic cohort therefore comprised 162 participants, with equal representation of G3, G4, and G5 and complete BRS data available for all included participants. The pooled mean age was 42.97 ± 12.76 years, and 128 participants (79.0%) were male. Age, sex distribution, BMI, and hemoglobin were comparable across categories. Both SBP and DBP differed significantly, with higher measured values in the later CKD categories. The expected category-wise decrease in eGFR and increase in serum creatinine were observed, as shown in Table 2.

**Table 2:** Category-specific demographic, hemodynamic, and renal characteristics Values are presented as mean ± standard deviation or number (percentage). Continuous variables were compared using Welch analysis of variance because equality of group variances could not be assumed. Sex distribution was compared using Pearson’s chi-square test. The test statistic and corresponding two-sided p-value are reported for each variable. For sex, the chi-square statistic, degrees of freedom, p-value, and Cramer’s V refer to the overall male-female distribution across CKD categories. BMI: body mass index; BP: blood pressure; CKD: chronic kidney disease; DBP: diastolic blood pressure; eGFR: estimated glomerular filtration rate; SBP: systolic blood pressure; SD: standard deviation

Variable	Statistic or category	G3 ( n= 54)	G4 (n = 54)	G5 (n = 54)	Test statistic	p-value
Age (years)	Mean ± SD	43.72 ± 13.06	44.30 ± 11.93	40.89 ± 13.24	Welch F = 1.079	0.344
Sex	Male, n (%)	43 (79.6%)	39 (72.2%)	46 (85.2%)	χ² = 2.755; df = 2; Cramer’s V = 0.130	0.252
	Female, n (%)	11 (20.4%)	15 (27.8%)	8 (14.8%)		
BMI (kg/m²)	Mean ± SD	23.94 ± 3.04	24.21 ± 3.26	23.70 ± 2.88	Welch F = 0.369	0.689
Resting BP	SBP, mmHg	139.07 ± 21.39	149.81 ± 20.05	159.46 ± 24.60	Welch F = 10.652	<0.001
	DBP, mmHg	91.11 ± 10.76	93.52 ± 9.74	97.20 ± 9.42	Welch F = 5.065	0.008
Renal profile	eGFR, mL/min/1.73 m²	42.77 ± 9.58	21.49 ± 5.42	8.60 ± 2.08	Welch F = 428.396	<0.001
	Hemoglobin, g/dL	11.60 ± 2.04	11.19 ± 1.85	11.25 ± 2.24	Welch F = 0.656	0.515
	Serum creatinine, mg/dL	1.91 ± 0.24	3.42 ± 0.78	7.57 ± 1.58	Welch F = 412.769	<0.001

All-sequence baroreflex sensitivity

**Figure 2:**
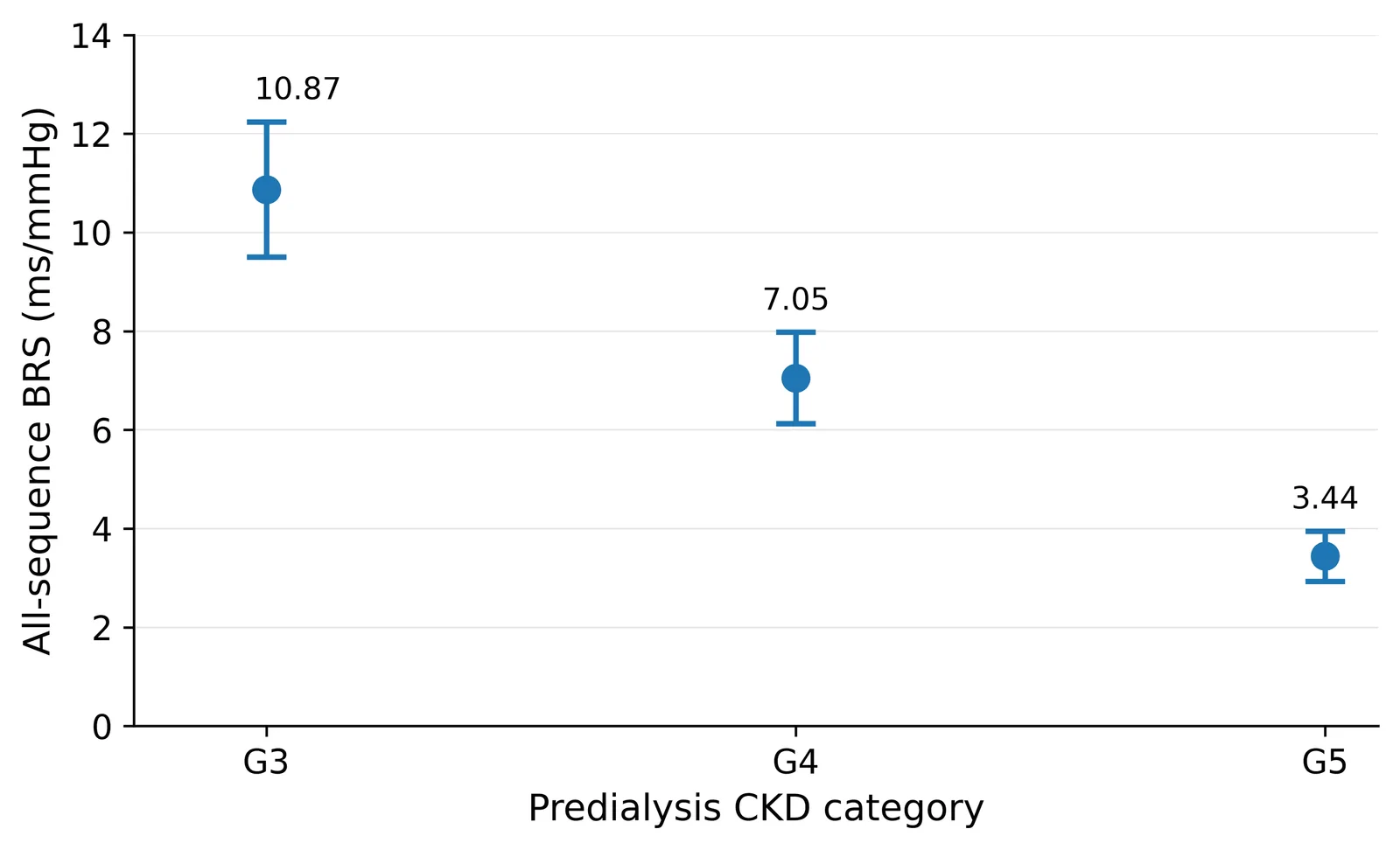
All-sequence baroreflex sensitivity across predialysis chronic kidney disease categories Points represent group means and vertical error bars represent 95% confidence intervals. All three Games-Howell pairwise comparisons were statistically significant (adjusted p < 0.001). BRS: baroreflex sensitivity; CKD: chronic kidney disease

All-sequence BRS was lower across successively higher CKD categories. The mean BRS was 10.87 ± 5.02 ms/mmHg in G3, 7.05 ± 3.39 ms/mmHg in G4, and 3.44 ± 1.86 ms/mmHg in G5. BRS was approximately normally distributed within each category (Shapiro-Wilk p = 0.840, 0.479, and 0.142, respectively), but Levene's test demonstrated unequal variances (p < 0.001). The primary comparison therefore used Welch analysis of variance and was statistically significant (F(2,91.98) = 65.22, p < 0.001). The conventional descriptive eta-squared estimate was 0.412, indicating a large unadjusted between-category effect (Table 3, Figure 2).

**Table 3:** All-sequence baroreflex sensitivity across predialysis CKD categories Values are mean ± SD with 95% CIs. The overall comparison used Welch analysis of variance because Levene's test indicated unequal variances. η² was calculated conventionally as SS_between_/SS_total_ and is presented as a descriptive effect-size estimate; the inferential group comparison was based on Welch analysis of variance (ANOVA). BRS: baroreflex sensitivity; CI: confidence interval; CKD: chronic kidney disease; SD: standard deviation

Category	n	Mean ± SD (ms/mmHg)	95% CI	Welch F (df)	p-value	Descriptive η²
G3	54	10.87 ± 5.02	9.50-12.24	65.22 (2,91.98)	<0.001	0.412
G4	54	7.05 ± 3.39	6.13-7.98			
G5	54	3.44 ± 1.86	2.93-3.95			

Games-Howell comparisons confirmed lower BRS at each successively higher CKD category. The largest difference was between G3 and G5 (mean difference 7.43 ms/mmHg, adjusted 95% CI 5.68-9.18) (Table 4).

**Table 4:** Games-Howell pairwise comparisons for all-sequence baroreflex sensitivity Mean differences are expressed as the earlier CKD category minus the later category. Games-Howell testing was selected because group variances were unequal. BRS: baroreflex sensitivity; CI: confidence interval; CKD: chronic kidney disease; SE: standard error

Comparison	Mean difference (ms/mmHg)	SE	Games-Howell 95% CI	Adjusted p-value	Result
G3 vs. G4	3.81	0.82	1.85 to 5.78	<0.001	Significant
G3 vs. G5	7.43	0.73	5.68 to 9.18	<0.001	Significant
G4 vs. G5	3.61	0.53	2.36 to 4.87	<0.001	Significant

Multivariable sensitivity analysis

**Figure 3:**
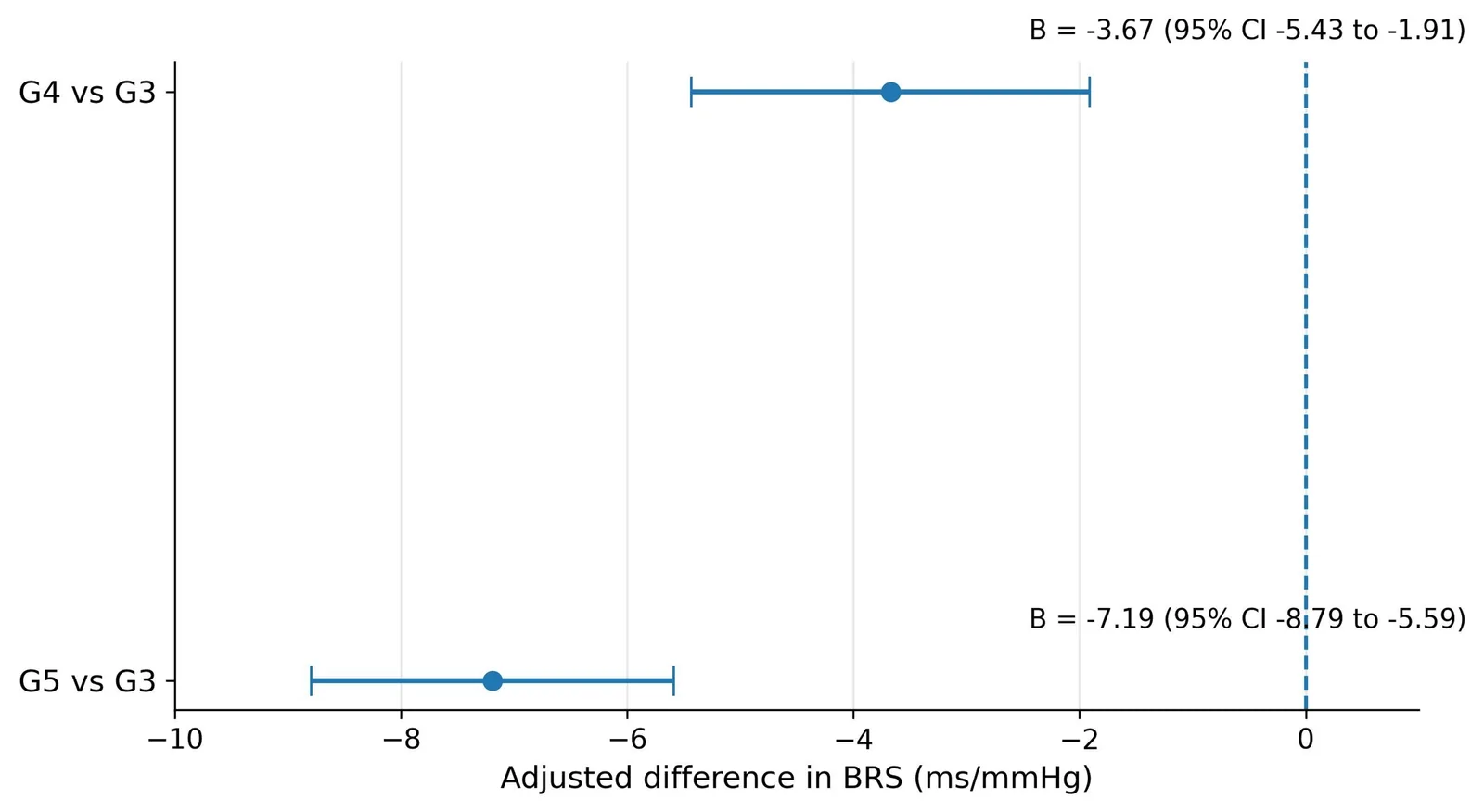
Fully adjusted CKD-category coefficients for all-sequence baroreflex sensitivity Points represent unstandardized coefficients from Model 2, and horizontal bars represent HC3 robust 95% confidence intervals. G3 was the reference category. The model adjusted for age, sex, body mass index, and resting systolic blood pressure. Negative coefficients indicate lower BRS relative to G3. BMI: body mass index; BRS: baroreflex sensitivity; CI: confidence interval; CKD: chronic kidney disease; HC3: heteroscedasticity-consistent standard error type 3; SBP: systolic blood pressure

Both regression models were statistically significant. Model 1, adjusted for age, sex, and BMI, explained 42.1% of the variance in BRS (R² = 0.421; adjusted R² = 0.403; robust F(5,156) = 26.35, p < 0.001). Compared with G3, the adjusted BRS was lower by 3.82 ms/mmHg in G4 and by 7.48 ms/mmHg in G5. After resting SBP was added in Model 2, the association remained significant: BRS was lower by 3.67 ms/mmHg in G4 and by 7.19 ms/mmHg in G5 relative to G3. Model 2 explained 42.6% of the variance (R² = 0.426; adjusted R² = 0.403; robust F(6,155) = 21.50, p < 0.001). Age, sex, BMI, and resting SBP were not individually significant. Variance inflation factors ranged from 1.03 to 1.54, indicating no meaningful multicollinearity (Table 5). The fully adjusted CKD-category coefficients and HC3 robust 95% CIs are displayed in Figure 3.

**Table 5:** Multivariable linear regression sensitivity analysis of all-sequence baroreflex sensitivity Model 1 adjusted for age, sex, and BMI. Model 2 additionally adjusted for resting SBP. G3 and female sex were the reference categories. Values are unstandardized regression coefficients with 95% CIs calculated using HC3 heteroscedasticity-consistent standard errors. BMI: body mass index; BRS: baroreflex sensitivity; CI: confidence interval; CKD: chronic kidney disease; SBP: systolic blood pressure

Predictor	Model 1 B (95% CI)	p-value	Model 2 B (95% CI)	p-value
G4 vs. G3	-3.82 (-5.51 to -2.13)	<0.001	-3.67 (-5.43 to -1.91)	<0.001
G5 vs. G3	-7.48 (-8.92 to -6.04)	<0.001	-7.19 (-8.79 to -5.59)	<0.001
Male vs. female	0.19 (-1.25 to 1.62)	0.800	0.19 (-1.24 to 1.63)	0.790
Age, per year	-0.02 (-0.07 to 0.02)	0.323	-0.02 (-0.07 to 0.02)	0.341
BMI, kg/m²	0.13 (-0.08 to 0.34)	0.231	0.13 (-0.08 to 0.33)	0.230
Resting SBP, per mmHg	Not included	Not included	-0.01 (-0.04 to 0.02)	0.362

## Discussion

The principal finding was a marked category-wise difference in spontaneous all-sequence BRS across three consecutive predialysis CKD categories. The mean BRS was approximately 35% lower in G4 than in G3 and approximately 68% lower in G5 than in G3. The effect size was large, and all pairwise comparisons remained significant after Games-Howell adjustment. In the post-hoc multivariable sensitivity analysis, the category association persisted after adjustment for age, sex, BMI, and resting SBP. The addition of SBP attenuated the G4 and G5 coefficients only modestly, and SBP itself was not significantly associated with BRS in the fully adjusted model. These findings indicate that measured differences in resting systolic pressure did not fully account for the observed BRS gradient. Because the study was cross-sectional and the adjusted analysis was post hoc, the findings should still be interpreted as associations rather than evidence of within-person progression or causality.

Bavanandan et al. evaluated 105 nondialysis CKD patients and found that lower BRS was related to reduced glomerular filtration rate, older age, and higher clinic systolic pressure [6]. Lacy et al. similarly associated reduced renal function with impaired BRS and reduced vascular compliance [7]. Lal et al. reported reduced spontaneous BRS in predialysis CKD [8]. The present study extends these observations by providing a balanced comparison across G3, G4, and G5 under a uniform five-minute recording protocol.

Martinez-Alanis et al. compared sequence-derived BRS in 18 patients with end-stage renal disease and 22 healthy volunteers using 15-minute supine and active-standing recordings with signal delays of zero to five beats. BRS was significantly lower in the end-stage renal disease group, and the delayed-sequence approach increased the number of accepted sequences while reducing estimation error [11]. Sabino-Carvalho et al. examined a complementary component of baroreflex physiology in 18 patients with CKD stages III-IV and 13 controls. Sympathetic BRS was attenuated in CKD (-1.34 ± 0.59 versus -2.91 ± 1.09 bursts per 100 heartbeats per mmHg; p = 0.001) and was associated with eGFR (r = 0.69, p < 0.001), supporting impairment of the neural sympathetic baroreflex arc in nondialysis CKD [12].

Theodorakopoulou et al. evaluated 68 patients receiving hemodialysis or peritoneal dialysis using a cross-correlation method at rest and during mental arithmetic, orthostatic, and handgrip testing. Resting BRS was 4.41 ± 3.16 ms/mmHg in the hemodialysis group and 5.89 ± 8.21 ms/mmHg in the peritoneal dialysis group, with no significant difference between modalities; BRS responses during stress testing were likewise similar [13]. The mean BRS of 3.44 ± 1.86 ms/mmHg observed in the present G5 predialysis group is directionally consistent with marked baroreflex impairment in advanced kidney disease. Direct numerical comparison is nevertheless inappropriate because the populations, recording conditions, and analytical methods differed.

Chesterton et al. reported an association between reduced BRS, vascular calcification, and arterial stiffness in CKD [9]. Charytan et al. demonstrated depressed BRS in individuals with both CKD and heart failure compared with those who had heart failure and preserved kidney function [10]. Johansson et al. subsequently reported that baroreflex effectiveness and BRS predicted all-cause mortality and sudden death in hypertensive chronic renal failure [18]. The present study did not assess clinical outcomes and therefore does not provide prognostic validation.

Several mechanisms may contribute to the observed category-wise reduction in BRS. Renal afferent activation, renin-angiotensin-aldosterone system activity, volume expansion, oxidative stress, impaired nitric oxide bioavailability, vascular remodeling, and uremic toxins can promote sympathetic excitation and reduce arterial compliance. Parasympathetic withdrawal and uremic autonomic neuropathy may further diminish rapid cardiac interval responses to pressure fluctuations [4,9,15]. These mechanisms are biologically compatible with progressively impaired baroreflex function at lower levels of kidney function, although causal pathways cannot be established by the present cross-sectional design.

Autonomic abnormalities may be partly modifiable. Kaur et al. demonstrated improvement in BRS after renal transplantation in parallel with improvement in central arterial stiffness [14]. Gao et al. demonstrated the reproducibility of several BRS methods in end-stage renal disease [19], and Gerhardt et al. also reported improved baroreceptor sensitivity after transplantation [20]. Together, these findings support prospective evaluation of BRS as a physiological research endpoint, but they do not establish clinical decision thresholds.

eGFR classifies renal severity but does not directly measure dynamic cardiovascular reflex control. Spontaneous BRS can be derived noninvasively from simultaneous short-term blood pressure and electrocardiographic recordings. The category gradient and its persistence in the adjusted sensitivity analysis suggest that BRS may help characterize cardiovascular autonomic impairment in predialysis CKD beyond measured differences in age, sex, BMI, and resting systolic pressure. At present, however, BRS should be regarded as a research measure rather than a validated diagnostic, prognostic, or treatment-monitoring tool. Although the between-category differences observed in this study were statistically large, validated clinical thresholds for spontaneous BRS in predialysis CKD have not been established, and the present study did not evaluate cardiovascular events or mortality. The findings should therefore be interpreted as physiological associations rather than evidence supporting clinical risk stratification.

Strengths and limitations

Strengths include stage-stratified consecutive recruitment with equal representation of G3, G4, and G5; confirmation of CKD chronicity; exclusion of major known nonrenal autonomic confounders; simultaneous beat-to-beat electrocardiographic and arterial-pressure recording; a confirmed five-minute stationary analysis segment; use of Nevrokard BRS version 6.3.0; a clearly defined primary outcome; complete BRS data in the prespecified cohort; appropriate assumption-based statistical methods; multiplicity-adjusted pairwise comparisons; effect-size reporting; and a post-hoc multivariable sensitivity analysis using HC3 robust standard errors.

Several limitations require consideration. The cross-sectional design prevents causal or temporal inference, and the absence of a healthy control group limits interpretation to category-wise differences within CKD. Albuminuria categories and the underlying etiologies of CKD were not systematically captured in the analytic dataset and therefore could not be reliably reported or incorporated into the statistical analysis. Consequently, complete KDIGO cause-GFR-albuminuria characterization could not be examined. Consecutive recruitment within fixed category quotas supports balanced comparison but does not estimate the natural distribution of CKD categories in the outpatient population. The exclusion of participants with diagnosed hypertension, diabetes mellitus, established cardiovascular or cerebrovascular disease, and medications known to materially influence autonomic measurements reduced important nonrenal influences on BRS and strengthened internal physiological comparability. However, because these conditions are common in routine CKD practice, the study population represents a selected predialysis CKD cohort and the findings should not be generalized directly to the broader CKD population, particularly patients with multiple cardiovascular or metabolic comorbidities. Other comorbid conditions were not systematically characterized in the analytic dataset and therefore could not be comprehensively reported or adjusted for. Generalizability is further limited by the single-center design, the age range of 18-65 years, and the predominance of male participants. Although recruitment continued consecutively within each CKD category until the prespecified number of analyzable participants was reached, complete screening logs documenting all patients assessed before enrolment, reasons for pre-enrolment exclusion, and technically unsuccessful recordings were not retained in a form permitting reliable retrospective reconstruction. Consequently, the proportion of screened patients who ultimately entered the analytic cohort cannot be reported, and selection related to recruitment or technical recording success cannot be fully excluded.

Although participants with a documented pre-existing diagnosis of hypertension or current antihypertensive treatment were excluded, resting systolic and diastolic pressures measured during the standardized study visit were higher in the later CKD categories. An isolated elevated study-visit measurement was not used as an exclusion criterion in the absence of a prior diagnosis or treatment history. The adjusted sensitivity analysis showed that the CKD-category coefficients remained significant after inclusion of resting systolic pressure, but this does not eliminate residual confounding. Systolic pressure may also lie partly on the biological pathway linking more advanced CKD to impaired baroreflex function, so adjustment may remove part of the CKD-related effect. Volume status, anemia, mineral-bone abnormalities, inflammation, and ongoing renal treatment were not fully characterized and may also have influenced BRS. Respiratory frequency was not objectively monitored or included in the adjusted analysis. Because spontaneous respiration can influence beat-to-beat cardiovascular variability and BRS estimates, unmeasured differences in breathing pattern across CKD categories may have contributed to the observed values. Resting heart rate and mean R-R interval were not included in the analytic dataset for category-wise comparison or multivariable adjustment. Because spontaneous cardiac BRS may be influenced by the prevailing cardiac interval, residual physiological confounding related to baseline R-R interval cannot be excluded.

Absolute BRS values are method-dependent. The present study used the spontaneous cardiac sequence method in Nevrokard BRS version 6.3.0 applied to a five-minute resting segment. Martinez-Alanis et al. used a delayed-sequence method with longer recordings, Sabino-Carvalho et al. quantified sympathetic BRS from muscle sympathetic nerve activity and diastolic pressure, and Theodorakopoulou et al. used a cross-correlation method during rest and stress testing [11-13]. Consequently, BRS estimates obtained using different physiological arcs, recording durations, lag structures, sequence thresholds, or analytical algorithms should not be treated as directly interchangeable. Recording duration can influence the number and stability of spontaneous baroreflex sequences available for analysis, while lag structure, sequence-length requirements, minimum systolic-pressure and R-R interval changes, and correlation thresholds determine which sequences are accepted and can therefore affect the resulting absolute BRS estimate. Accordingly, absolute BRS values should be interpreted in the context of the specific acquisition and analytical protocol used rather than compared directly across substantially different methodologies.

## Conclusions

Spontaneous all-sequence BRS was substantially lower across successively higher predialysis CKD categories, with significant differences between G3, G4, and G5. The association remained significant in a post-hoc sensitivity analysis adjusted for age, sex, BMI, and resting SBP, indicating that the observed category gradient was not fully explained by these measured covariates. Because the study was cross-sectional and did not evaluate clinical outcomes, the results do not establish temporal progression, causality, or prognostic utility. Prospective longitudinal studies incorporating complete KDIGO cause-GFR-albuminuria characterization, prespecified multivariable analysis, and cardiovascular outcome follow-up are required before BRS can be recommended for clinical risk stratification or serial treatment monitoring.
